# When unusual corpus alienum invade the urethra in schizophrenia patients: A case report

**DOI:** 10.1016/j.ijscr.2025.110920

**Published:** 2025-01-21

**Authors:** Nadya Rahmatika, Soetojo Wirjopranoto, Bagus Wibowo Soetojo, Yufi Aulia Azmi, Antonius Galih Pranesdha Putra, Kevin Muliawan Soetanto

**Affiliations:** a Faculty of Medicine, Wijaya Kusuma University, Surabaya, Indonesia; b Department of Urology, Faculty of Medicine Universitas Airlangga – Dr. Soetomo General Academic Hospital, Surabaya, Indonesia; c Department of Orthopaedic and Traumatology, Faculty of Medicine, Universitas Airlangga – Dr. Soetomo General Academic Hospital, Indonesia; d Department of Health Sciences, University of Groningen, University Medical Center Groningen, Groningen, the Netherlands; e Department of Immunology, Faculty of Medicine Siriraj Hospital, Mahidol University, Bangkok, Thailand

**Keywords:** Urethra, Corpus alienum, Schizophrenia, Case report, Tweezers extraction

## Abstract

**Introduction:**

The presence of foreign or unexpected external objects in the urinary tract, including the urethra, is a rare case. This case is a challenge for patients with schizophrenia. This case report presents when the unusual corpus alienum invades the urethra in schizophrenia patients.

**Case presentation:**

A 54-year-old man was referred to the emergency room with corpus alienum tweezers inside the urethra. The patient had a history of schizophrenia 30 years ago and had not been routinely treated for the past year. From the physical examination, it was found that the tip of the tweezers penetrated out of the penile gland, and the patient could still urinate without bloody fluid. A pelvic X-ray shows tweezers in the urethra. The results of the urethrography showed a corpus alienum with both ends penetrating the penile gland. There is no clear contrast image visible filling the anterior urethra and a contrasting backflow from the urethra to the hole where the end of the corpus alienum comes out. Management of schizophrenia patients, given haloperidol tablets 2.5 mg/12 h and regular check-ups at psychiatric clinics.

**Discussion:**

Treatment is based on two steps: foreign body extraction and psychiatric treatment for mental illness. Patients need routine treatment, and haloperidol is one of the drugs that can be used.

**Conclusion:**

Extraction management of corpus alienum urethra must be carried out immediately to prevent complications. Comprehensive management is needed if there are other diseases, such as schizophrenia, in these patients to prevent recurrence.

## Highlights

• Management for unusual corpus alienum invade the urethra in schizophrenia patient
• Comprehensive management urology and psychiatry
• Routine of follow up for patient condition, especially for the schizophrenia condition

## 1 Introduction

The presence of external objects entering the human body can cause immunity problems. This case is called a ‘foreign body’ or Corpus Alienum in Latin [1]. The problem was also found to be a foreign object entering the urinary tract. Foreign bodies in the urinary tract are becoming a topic of interest among urologists and surgeons [2]. Previous research has identified examples of foreign objects that enter and are reported in the bladder, including the presence of cables, canes, medical devices, contraceptives, pencils, and other objects [3].

An uncommon urologic emergency is foreign bodies in the lower genitourinary tract [4]. Several processes, including self-insertion, iatrogenic causes, and damage from nearby organs, can allow foreign bodies to enter the bladder. Self-insertion is one of the main causes of the rise in intravesical foreign bodies [3]. Symptoms including dysuria, hematuria, increased frequency and urgency of urine, and lower abdominal and pelvic pain might be caused by foreign bodies in the lower urinary tract. Furthermore, the nature, size, form, movement, location, and duration of occupancy of the FBs all influence these symptoms [5].

The presence of a foreign body in the urethral duct can cause injury. Urethral injuries are a relatively rare medical condition. Urethral injuries are never life-threatening, but if left untreated can lead to significant morbidity [6]. Cases of injury can be experienced by patients with comorbidities such as psychological conditions. Psychological conditions can cause patients to do unexpected things, such as in patients with schizophrenia. Inserting a foreign object is a common practice for self-harm or other purposes in patients with chronic psychosis [7]. Schizophrenia affects people of all age groups [8]. Non-adherence to medication can exacerbate mental illness and create new problems therefore mental health services should improve treatment strategies to prevent harmful behaviors [9]. Treatment of patients with mental disorders accompanied by other illnesses is a challenge for health facilities [10]. Reports of this case have been reported in line with the SCARE Guidelines [11]. This case report presents when the unusual corpus alienum invades the urethra in schizophrenia patients.

## 2 Case presentation

**Fig. 1:**
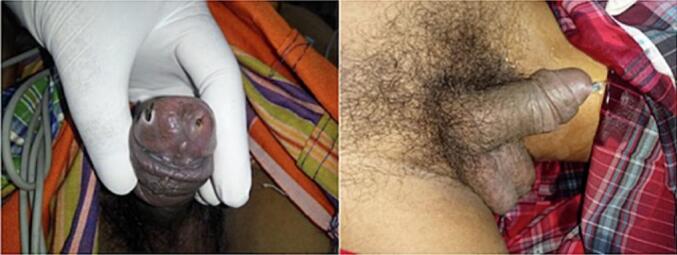
Clinical picture of the patient.

**Fig. 2:**
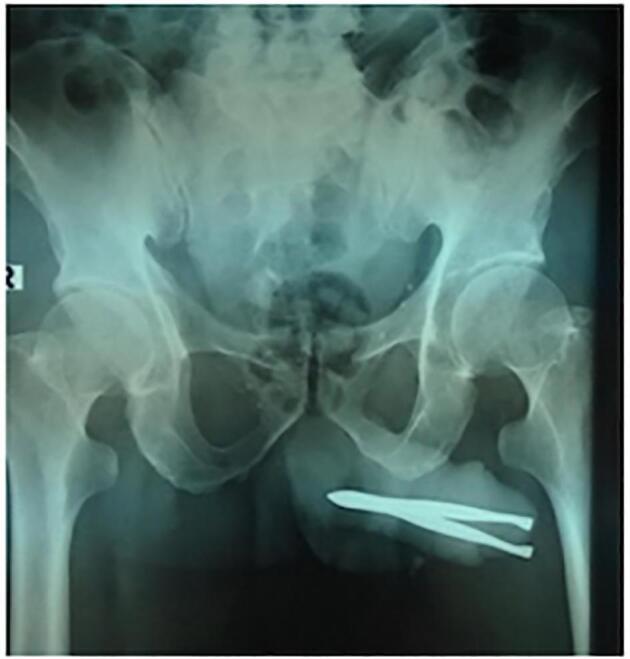
Pelvic X-ray, tweezers in the urethra.

**Fig. 3:**
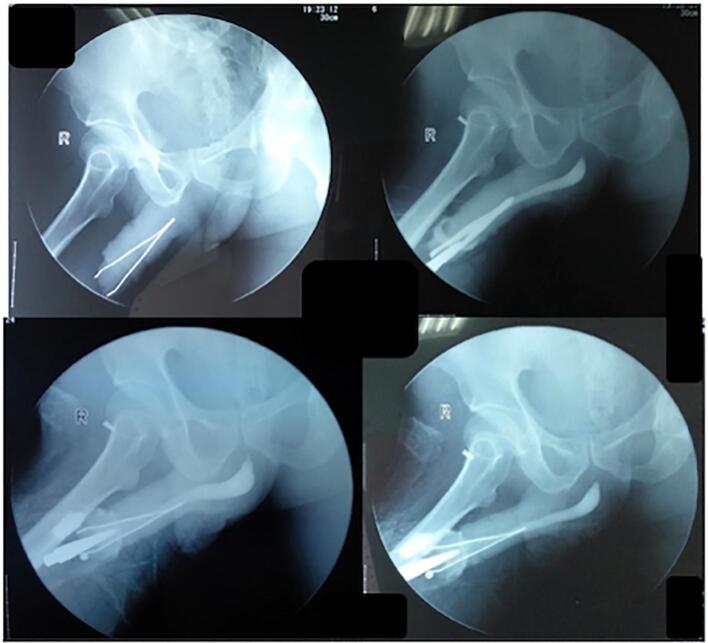
Urethrography results.

A 54-year-old man was referred to the emergency room (ER) with corpus alienum tweezers inside the urethra. His family already knew about the tweezers in the urethra in the morning before entering the hospital; when the patient urinates, he screams in pain with a visual analog score (VAS 5). The patient is unemployed, lives alone at home, is unmarried, and has a history of schizophrenia since 30 years ago. A psychiatrist has not routinely treated patients for schizophrenia during the past year. From the physical examination, there are typical vital signs; It was found that the tip of the tweezers penetrated out of the penile gland, and the patient could still urinate without bloody fluid (Fig. 1). Laboratory results are expected. Laboratory results were performed to assess the presence of white blood cells or red blood cells seen in the hematuria analysis. The results showed that there were these signs. Urethral foreign bodies can cause urinary tract infections. A pelvic X-ray shows tweezers in the urethra (Fig. 2). The results of the urethrography showed an alien corpus with both ends penetrating the penile gland, causing partial obstruction of the anterior urethra; There is no clear contrast image visible filling the anterior urethra, a contrasting backflow from the urethra to the opening where the end of the alienum corpus exits (Fig. 3). Therefore, we do tweezer extraction; the base of the penis is pressed to prevent the tweezers from sliding deeper into the urethra. The external urethral meatus is slightly sliced on the dorsal side to facilitate tweezer extraction. The tip of the tweezers is pushed slightly and comes out of the wound in the urethra; then the tweezers are removed. Tweezer extraction was performed under anesthesia. The X-ray image after the tweezer extraction is related to the financing of national universal health coverage in Indonesia. In addition, there was no other foreign body image in the urethra. A pelvic X-ray was sufficient at the beginning. No bleeding was found. A 0.3 cm puncture wound in the urethra is sutured with a plain catgut 3.0. The 16-fr silicone catheter is inserted and removed after 30 days. There are no complaints related to urination. Management of schizophrenia patients, given haloperidol tablets 2.5 mg/12 h and regular check-ups at psychiatric clinics; After 2 months of routine treatment and psychiatric clinic examinations, patients can be invited to communicate.

## 3 Discussion

This case report shows the need for the extraction of the corpus alienum urethra to prevent complications on the urethra. Urethral injuries are a relatively rare medical condition Doctors classically use several anatomical landmarks in the evaluation, classification, and management of urethral injuries, which are especially important among male patients. Urethral injuries put patients at risk of future morbidity. Early complications that may occur are centered around secondary infections, including the formation of abscesses. Early complications can be found around secondary infections, including the formation of abscesses. Final complications can include urethral stricture and stenosis, fibrosis, removal of the urethral lumen, formation of urethrocutaneous fistulas, urinary incontinence, and erectile dysfunction [12]. In these cases, no initial complications were found that could likely lead to a poor prognosis.

In this case, the patient had tweezer extraction. Urological trauma can cause a well-known urethral injury with a variety of management recommendations. Retrograde urethrogram remains the preferred initial diagnostic modality for evaluating suspected urethral injury [6]. In this case, an examination was carried out Urethrography. Diagnosis can sometimes be difficult if it is reported late or if the patient is not cooperating. Treatment is based on two steps: the extraction of the foreign body by endoscopy or open surgery and the psychiatric treatment for the mental illness [7]. In other studies, several diagnostic procedures such as X-rays, CT scans and open cystotomy were explained, as well as digital rectal examination (DRE). The management also varies based on the case experienced. This is by Table 1 which shows the related cases Corpus Alienum in the urethra.

**Table 1:** Cases related to corpus alienum in the urethra.

Author, year	Patient characteristics	Comorbidities	Types of foreign objects and their location	Diagnostic procedure	Suggestion
Fotovat et al. (2023) [13]	A 27-year-old man	Psychotic disorders	Inserting the pen ink chamber into the urethra	X-rays, CT scans, and open cystotomy	Timely surgery to prevent urine retention and psychological support.
Anass et al. (2024) [14]	A 26-year-old man	schizophrenia	Self-inserting a 65 cm metal cable into his urethra	Penile palpation combined with digital rectal examination (DRE)	Surgical removal, prophylactic antibiotics, and urethra are closed with a silicone catheter. The patient's psychiatric condition requires early catheter removal. Patients are referred to psychiatric services for ongoing care.
Trigui et al. (2023) [15]	A 23-year-old man	schizophrenia	Self-inserting two nails into his scrotum	Palpation, A plain X-ray	Psychiatric evaluation is mandatory to detect an underlying mental disorder and to avoid repeat auto-insertion.
Ouskri et al. (2025) [16]	A 37-year-old man	schizophrenia	Calcified bladder foreign bodies	Clinical examination, pelvic ultrasound, plain abdominal X-ray (AUSP), CT scan	The management requires accurate diagnosis based on modern imaging techniques and tailored surgical intervention. Postoperative psychiatric care is essential to prevent high-risk behaviors and improve long-term outcomes.

Comprehensive management is needed when there are other diseases, such as schizophrenia in these patients to prevent recurrence. This patient had a history of schizophrenia dating back 30 years. The management of schizophrenia patients is given haloperidol tablets 2.5 mg/12 h and routine check-ups at the psychiatric clinic, after 2 months of undergoing routine treatment and psychiatric clinic examinations, patients can be communicated. Medication adherence in schizophrenia patients is essential. Non-adherence to treatment can worsen mental illness and make treatment more challenging. Mental health services should strive to improve treatment adherence strategies and offer social support to prevent harmful behaviors [9]. Haloperidol, a typical first-generation antipsychotic, is commonly used worldwide to block dopamine D2 receptors in the brain and exert its antipsychotic action [17]. Haloperidol, one of the first-generation antipsychotic drugs, is effective in the treatment of schizophrenia but can have adverse side effects [18].

## 4 Conclusion

The management of the extraction of corpus alienum urethra must be carried out immediately to prevent complications. Comprehensive management is needed if there are other diseases, such as schizophrenia in these patients to prevent recurrence.

## Informed consent

The data used and reported have been authorized by the patient.

## Ethical approval

Ethical approval for this study was provided by Health Research Ethics Committee of Dr. Soetomo General-Academic Hospital, Surabaya,

## Guarantor

Soetojo Wirjopranoto

## Research registration number

Not required.

## Funding

No financial contributions were made by the authors to this study.

## Additional information

The information contained in this paper is personal.

## Author contribution

Nadya Rahmatika: Conceptualization, Data Curation, Writing-Original draft preparation

Bagus Wibowo Soetojo: Conceptualization, Data Curation, Writing-Original draft preparation

Soetojo Wirjopranoto: Conceptualization, Methodology, Data Curation, Investigation, Writing-Original draft preparation, Supervision, Validation

Yufi Aulia Azmi: Conceptualization, Methodology, Data Curation, Investigation, Writing-Original draft preparation, Supervision, Validation

Antonius Galih Pranesdha Putra: Writing-Original draft preparation, Writing-Reviewing, and Editing

Kevin Muliawan Soetanto: Writing-Original draft preparation, Writing-Reviewing, and Editing

## Conflict of interest statement

The author stated that there was no conflict of interest.

## Data Availability

The research outlined in the article does not make use of any data.
